# Luminescence Vapochromism of a Cu(I)/Na Phosphane Iodide Assembly Based on a Secondary‐Sphere Sodium Response

**DOI:** 10.1002/anie.6117838

**Published:** 2026-09-15

**Authors:** Henna Korhonen, Dorota Głosz, Mariia Beliaeva, Andrey Belyaev, Constance Lecourt, Adrien Schlachter, Karine Costuas, Guillaume Calvez, Robert Podgajny, Christophe Lescop, Igor O. Koshevoy

**Affiliations:** ^1^ Department of Chemistry and Sustainable Technology University of Eastern Finland Joensuu Finland; ^2^ Faculty of Chemistry Jagiellonian University in Krakow Kraków Poland; ^3^ ISCR (Institut Des Sciences Chimiques De Rennes) University of Rennes, INSA Rennes, CNRS Rennes France

**Keywords:** copper, heterometallic complexes, luminescence, phosphane ligands, vapochromism

## Abstract

Photoluminescent compounds showing distinct and selective optical response to external perturbation are essential components for sensing applications. Herein, we present a copper(I) iodide–sodium luminescent complex assembled with a hybrid phosphane‐phosphinate ligand. The photoemission of the tetrametallic {Cu_2_I_2_Na_2_} aggregate is consistent with the thermally activated delayed fluorescence that originates from the [CuI(phosphane)] motifs but strongly depends on the coordination environment of sodium ions completed by O‐donor solvent molecules, Na–π(phenyl) interactions, and/or Na–I bonds. The reversible removal and vapor‐phase addition of methanol, ethanol, water, and dimethoxyethane as Na‐bound ligands alter the solid‐state luminescence from blue (461 nm) to yellow (570 nm) with quantum yields reaching 0.73. Importantly, different photoemission responses to methanol (522 nm) and ethanol (492 nm) are complemented by preferred binding of methanol and its detection in up to 1:10 methanol:ethanol mixtures. Variations of luminescence among the studied solvates are attributed to the alterations of secondary sphere interactions involving sodium ions and illustrate a new approach to stimuli‐responsive metal complexes constructed from soft‐hard donor ligands.

## Graphical abstract

The luminescence of a heterometallic d‐s {Cu_2_I_2_Na_2_} phosphane‐phosphinate aggregate is regulated by an interplay of Na^+–^π/I^–^ contacts and reversible coordination of oxygen‐containing organic molecules to Na ions. Structural variations caused by the incoming guest lead to a wide and selective variation of the emission color and illustrate a new approach to responsive metal complexes constructed from soft‐hard donor ligands.

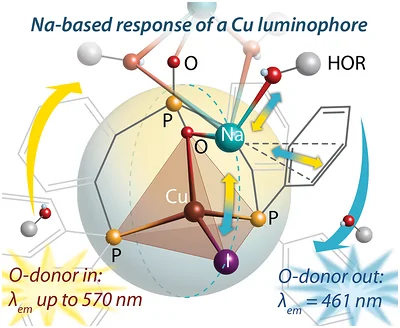

## 1 Introduction

Luminescent transition metal complexes showing reversible switches of the emission color in the solid state upon exposure to volatiles are intriguing molecular materials due to their potential applications in chemical sensing and optical monitoring devices [1, 2, 3]. Such vapor‐induced changes in photophysical properties commonly originate either from weak noncovalent interactions, direct coordination of the vapor guest molecules as ligands to metal centers, or a combination of these effects. Vapochromic behavior has been extensively studied among Au(I) and Pt(II) complexes, for which it is often associated with alterations of metal‐metal interactions [4, 5].

Cu(I) compounds form another actively investigated class of luminophores [6, 7, 8, 9]. Electronic transitions in molecular Cu(I) complexes, which account for light absorption and emission, have metal‐to‐ligand, ligand‐to‐ligand, and intraligand charge transfer (MLCT, L/XLCT, and ILCT) character. The optical response of these compounds to vapors and/or mechanical input, therefore, is primarily related to the perturbation of the ligand environment that often includes transitions between amorphous and crystalline phases or solvatomorphism [10, 11, 12, 13, 14, 15]. A common mechanism in this respect is the modulation of intra‐ and intermolecular π–π stacking or guest–π interactions involving aromatic fragments, which contribute to frontier molecular orbitals. For instance, the diphosphane pyridyl‐pyrazolate dicopper complex undergoes a reversible crystalline phase transition, triggered by the uptake of dichloromethane molecules that get entrapped between the pyridyl rings, resulting in the red‐shift of the emission from green to yellow [13]. A tetrametallic pyridyl‐tetrazolate congener shows selectively to acetone a blue shift of the emission (green→blue) related to a change of intramolecular pyridyl–phenyl π–π interactions [16]. The same group developed another approach, which relies on the use of ligands capable of hydrogen bonding with volatile molecules and/or counterions by means of a protic function, for example, non‐coordinated N–H groups of triazole‐containing diimines [17, 18] or diphosphanes [19, 20]. Their copper complexes exhibit multi‐stimuli responsive properties, where hydrogen bonds are broken under the external mechanical stimulation and reformed when exposed to vapors of organic solvents (CH_2_Cl_2_, methanol, acetone, etc.). An interesting case of a 1D coordination polymer composed of aqua‐bridged copper diphosphane dimers shows vapor‐dependent luminescence associated with the motion of solvent molecules along the channels of the crystalline lattice [21].

Distinct variations of the solid‐state emission can appear upon binding of the guest molecule to the copper center, resulting in the ligand substitution or a change of coordination number [12, 22, 23]. Thus, labile tetrahydrofuran in a weakly emissive cationic phosphane pyridine complex (*λ*_em_ = 457 nm, *Φ*_em_ = 0.02) is easily exchanged for N‐heterocyclic molecules. Vapor treatment leads to the modification of the ligand sphere and yields materials with sky blue (*λ*_em_ = 484 nm, *Φ*_em_ = 0.63 for methylpyridine), green (*λ*_em_ = 510 nm for pyrimidine), and orange (*λ*_em_ = 618 nm for pyrazine) luminescence [24]. A comparable effect was observed upon gas‐phase addition of N‐donors to a three‐coordinate complex [Cu(diphosphane)I] (*λ*_em_ = 497 nm, *Φ*_em_ = 0.02), which was converted into intensely luminescent tetracoordinate derivatives [Cu(diphosphane)I(L)] (*λ*_em_/*Φ*_em_ = 502 nm/0.67 and 573 nm/0.63 for L = pyridine or methylpyrazine, respectively) [25].

Furthermore, organic volatiles can evoke isomerization or conformational reorganization. Methanol vapors transform a binuclear blue emissive complex [Cu_2_(PPh_2_py)_3_(NCMe)]^2+^ with head‐to‐tail bound phosphano‐pyridines into a head‐to‐head linkage solvated isomer with green luminescence [26]. A bromide carbazole‐diphosphane copper(I) cluster was obtained in two polymorphs interconverted by hexane and acetonitrile vapors. Their green and yellow phosphorescence was attributed to different orientations of the organic ligand and the degree of structural distortion occurring in the excited state [27].

In this respect it is worth noting that hard donor O‐containing compounds (water, alcohols, ethers, aldehydes/ketones, etc.) have low affinity to copper(I) ions and rarely coordinate to this metal in a low oxidation state [21, 24]. The alterations of optical properties induced by oxygen‐containing organic vapors are typically not related to the interactions with the chromophore motif but rather correspond to non‐selective phase transitions resulting from co‐crystallization and/or hydrogen bonding [20, 28, 29].

New properties of coordination compounds can emerge from the use of mixed hard‐soft donor ligands, which allow for the assembly of both homo‐ and heterobimetallic species integrating coordination sites with different binding behavior. In particular, phosphanes decorated with anionic groups (amide/diketiminate, carboxylate, phenolate, and phosphide) serve as assembling units for the preparation of diverse photofunctional copper‐subgroup metal complexes [30, 31, 32, 33, 34, 35, 36, 37].

In our previous work we utilized a hybrid phosphane, bis(2‐(diphenylphosphaneyl)phenyl)phosphinic acid PPh_2_‐C_6_H_4_‐P(OOH)‐C_6_H_4_‐PPh_2_ = [***P* ^*3*^ *OO***]H, to prepare luminescent binuclear complexes of silver and gold, [M_2_(***P* ^*3*^ *OO***)_2_(H)*_n_*]*^n^* ^+^ (*n* = 1, 2) [38]. Their photophysical behavior significantly depends on protonation/deprotonation and hydrogen bonds involving the phosphinate R_2_POO– group (R = ‐C_6_H_4_‐PPh_2_). While the analogous copper(I) complex [Cu_2_(***P* ^*3*^ *OO***)_2_] appeared to be unstable, here we investigated the coordination chemistry of the sodium salt of the ligand, PPh_2_‐C_6_H_4_‐P(OONa)‐C_6_H_4_‐PPh_2_ = [***P* ^*3*^ *OO***]Na (Scheme 1), toward copper(I) iodide. The resulting copper/sodium iodide phosphane–phosphinate assembly shows intense solid‐state luminescence, which is governed by intramolecular sodium‐π and sodium‐iodide interactions and is switched by vapors of hard oxygen‐donor molecules (methanol, ethanol, and dimethoxyethane) reversibly bound to alkali metal ions.

**SCHEME 1:**
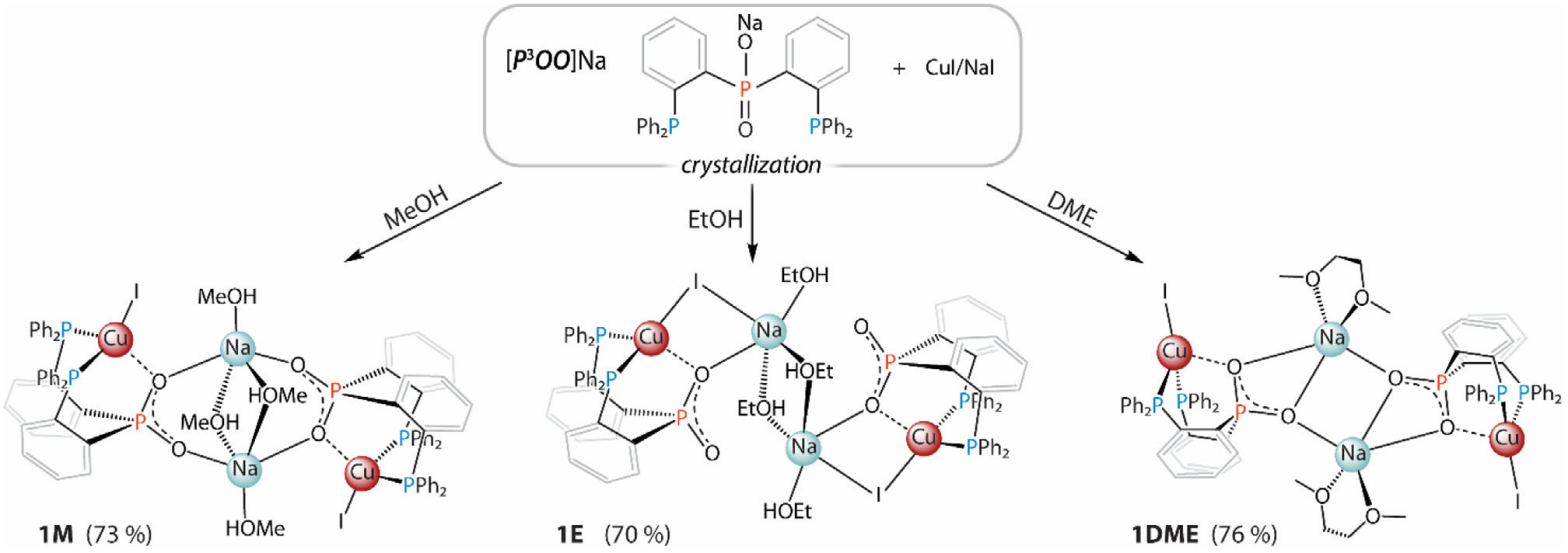
Preparation of solvates **1M**, **1E**, and **1DME** (see experimental details in the ESI).

## 2 Results and Discussion

Nearly colorless block crystals of complex [CuI(***P^3^OO***)Na(MeOH)_2_]_2_ (**1M**) showing bright green luminescence were isolated in good yield by a gas‐phase diffusion of diethyl ether into a methanol/toluene (4:1 v/v) solution containing equimolar amounts of copper(I) iodide, the ligand [***P* ^*3*^ *OO***]Na and sodium iodide (Scheme 1 and ESI). Using ethanol as a crystallization solvent afforded the solvate [CuI(***P^3^OO***)Na(EtOH)_2_]_2_ in a predominant form **1E_b**, showing sky‐blue emission, while a minor phase **1E_y**, can be distinguished by its yellow luminescence. The latter subsequently undergoes spontaneous transformation into the stable blue‐emitting material **1E_b** upon prolonged standing in a crystallization system. Using 1,2‐dimethoxyethane (DME) as a non‐protic ligand allowed the isolation of colorless crystals of the **1DME** modification, exhibiting yellowish luminescence. The results of *x*‐ray diffraction analysis are shown in Figures 1, S3–S5, the crystal and selected structural data are listed in Tables S1 and S2 [39].

**FIGURE 1:**
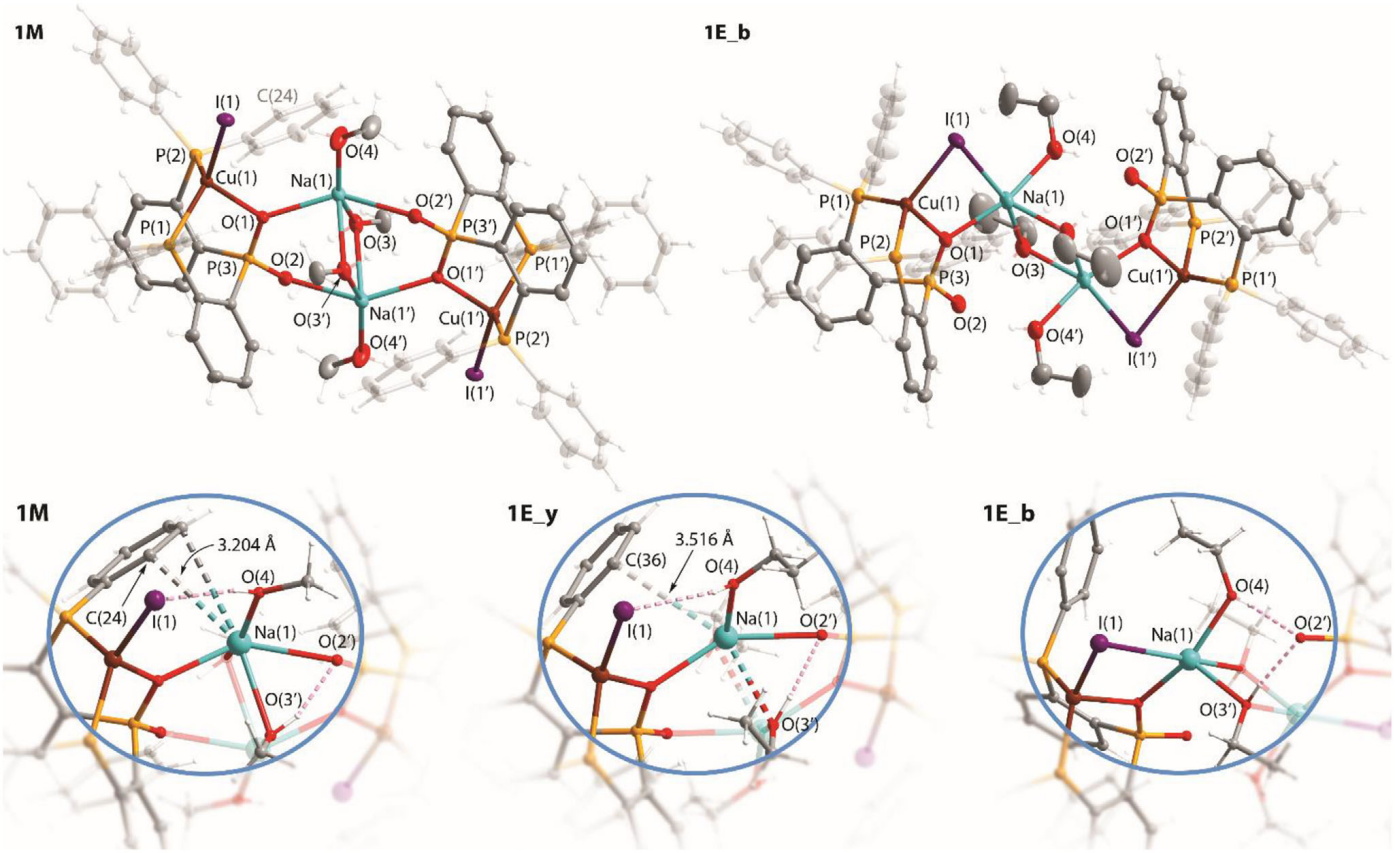
Top: molecular views of methanol (**1M**) and ethanol (**1E_b**) solvates (displacement ellipsoids are at the 50% probability level; disordered non‐coordinated crystallization ethanol in **1E_b** is omitted for clarity). Bottom: molecular fragments highlighting intramolecular I/O^…^H–O hydrogen bonding and Na–π interactions.

The examples of copper(I)–alkali metal heteronuclear compounds are not numerous [40, 41, 42, 43, 44]. In crystallographically characterized forms **1M**, **1E**, **1DME**, the molecular assemblies consist of two equivalent {CuI(***P^3^OO***)}^–^ blocks connected via sodium ions bound to oxygen atoms of the phosphinates. The ligand environment of alkali centers is further supplemented with O donors of alcohol/DME molecules. In **1M**/**1E**, alcohols are coordinated in terminal and bridging modes, the latter producing rhomboidal {Na_2_O_2_} motifs. A different configuration is observed for **1DME**, in which the DME ligands chelate sodium ions and the central {Na_2_O_2_} fragment is formed with oxygen atoms of POO^–^ residues. In both structurally very similar **1M** and **1E_y**, the ROH ligands (R = Me, Et) are further held by hydrogen bonding with iodides (*d* O(4)^…^I(1) are 3.493(1) and 3.592(2) Å, Table S2) and the phosphinate groups (Figure 1). The methanol solvate **1M** reveals non‐negligible Na–π(phenyl) interactions resulting in appreciable C^…^Na contacts of 3.204(1) and 3.432(2) Å (Figure 1) comparable to the reported examples of alkali cation–arene bonding [45, 46]. The metastable ethanol form **1E_y** features substantially longer C^…^Na distance exceeding 3.5 Å (Figure 1). In **1DME**, the shortest C_Ph_^…^Na separation exceeds 3.691 Å (Figure S4). The lack of Na–π interaction in **1E_y** probably drives the transition to **1E_b** modification, in which it is compensated by the Na–I bond (*d*_Na–I_ = 3.176(1) Å, Table S2). This coordination of the iodide ligand, bridging the copper and sodium ions, only slightly elongates the Cu–I bond in **1E_b** to 2.5833(4) Å compared to 2.5572(2)/2.5535(3) Å in **1M**/**1E_y** [42, 43, 47].

Neat crystals of methanol solvate **1M** undergo gradual exchange of methanol for water molecules when exposed to air at ambient conditions for several hours. The transformation is quantitative and proceeds in a single‐crystal‐to‐single‐crystal manner to give water solvate [CuI(***P^3^OO***)Na(H_2_O)_3_]_2_ (**1W**, Figure S5) with yellowish luminescence of lower intensity. **1W** has a non‐centrosymmetric arrangement in which sodium atoms are found in different coordination spheres.

In all structures, copper ions adopt typical pseudo‐tetrahedral geometry completed by two phosphane groups, the iodide, and O(PO) function. In contrast to sodium centers, the coordination spheres of copper have the same compositions and show relatively small deviations of conventional bond lengths and angles, which are comparable to those in congener copper(I) derivatives [11, 15, 25, 29, 48]. These structural observations purport that the differences in eye‐perceived photoemission of the titled materials primarily arise from alterations of the secondary sphere around Na ions and their subtle impact on the chromophore moiety.

In CD_2_Cl_2_:MeOD solution, the complex is stereochemically non‐rigid and does not retain its molecular structure, judging by broad and unresolved resonances in ^31^P{^1^H} NMR spectrum (see ESI), which indicates the coordination of phosphine groups to copper ions. Nevertheless, it provides no proof of the overall molecular integrity. As no appreciable luminescence was detected in solution, the photoluminescence properties were investigated for solid samples only.

To preserve the solvation environment of sodium ions, the photophysical measurements were carried out for powdered samples of **1M**/**1E** under MeOH/EtOH vapors; the identities of the studied phases were confirmed by PXRD analysis (Figures S6–S10). The material **1M** shows a structureless broad band with a maximum around 522 nm and a quantum yield *Φ*_em_ of 0.46 (Figure 2 and Table 1). Under ambient conditions, the emission of **1M** gradually red‐shifts to approximately 550 nm confirming the conversion to the water solvate **1W** (Figure S11). Time‐resolved measurements (Figure S12) provide a biexponentially fitted decay for **1M**, which gives an amplitude‐weighted average lifetime *τ*_av_ = 12.96 µs and the radiative rate *k*_r_ = 3.5^.^10^4^ s^–^^1^. At 80 K, the lifetime reveals a substantial growth to 109 µs that is indicative of two different excited states involved in the observed emission at ambient and cryogenic conditions (Figures S12 and S13). Such behavior is compatible with thermally activated delayed fluorescence (TADF), frequently encountered for luminescent copper(I) phosphane complexes usually associated with increasing participation of phosphorescence upon temperature decrease [8]. The time‐dependent density functional theory (TD‐DFT) calculations reveal that the nearly degenerate S_1_ and S_2_ states are formed from the transitions between the pairs of energetically close orbitals HOMO/HOMO – 1 and LUMO/LUMO + 1 (Figure S16 and Table S3). These states have an M + X to L (Cu‐I → π*) charge transfer character and are anticipated to dominate the room temperature emission in accordance with the TADF model. For comparison, ligand [***P* ^*3*^ *OO***]Na demonstrates prompt fluorescence at 297 K (Figures S12 and S14) that corresponds to an electronic density transfer from the phosphorus and oxygen atoms to the π* network of one of the phenyl groups (Table S3, Figure S15).

**FIGURE 2:**
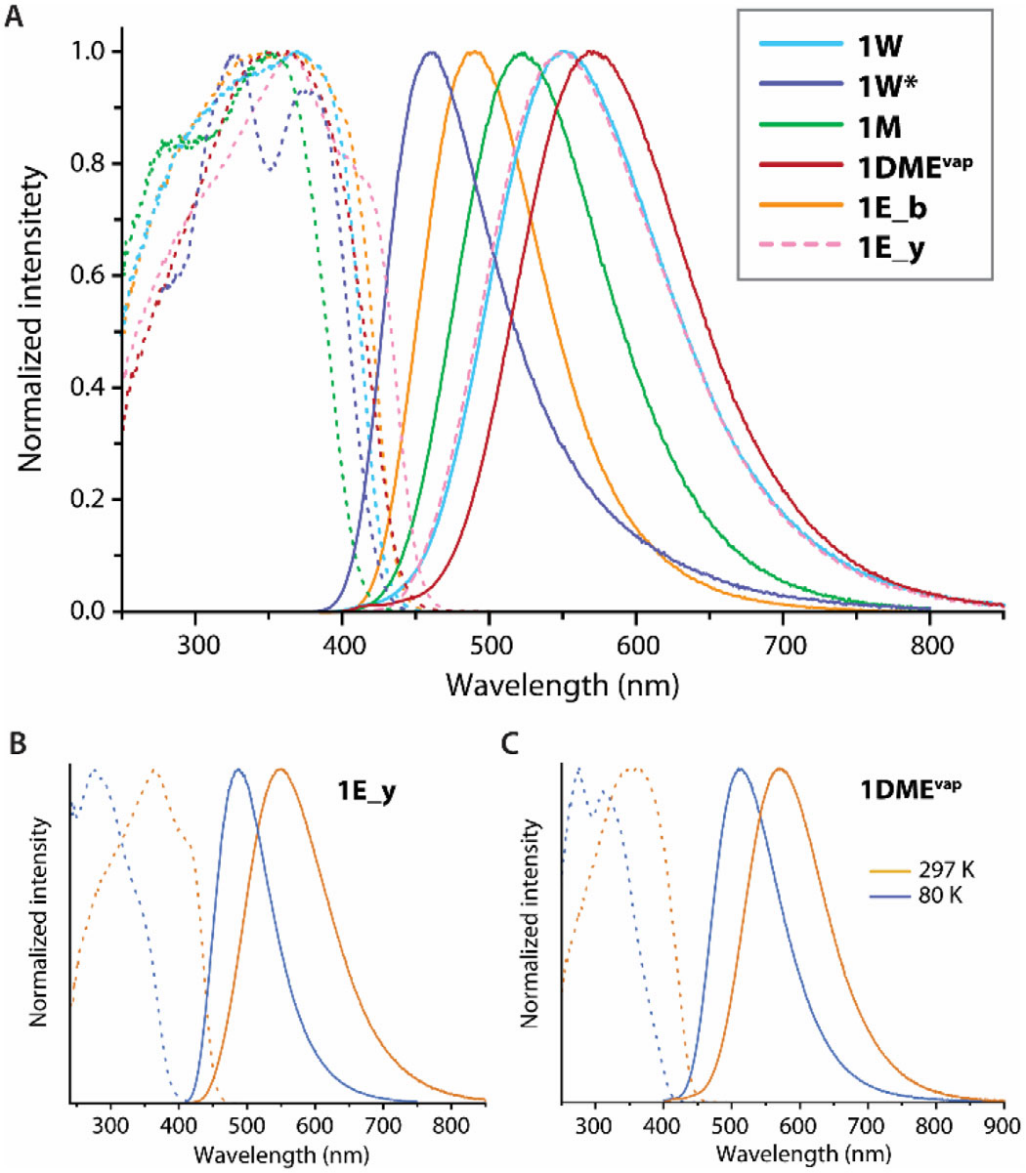
(A) normalized excitation and emission spectra of solid **1M**, **1E_b**/**1E_y**, **1** **W**/1W* and **1DME^vap^** at 297 K; (B, C) normalized excitation and emission spectra of **1E_y** and **1DME^vap^** at 297 and 80 K.

**TABLE 1:** Photophysical properties of the titled compounds in the solid state.

	297 K					80 K	
Compound	*λ*_em_, nm	*τ*_av,_ µs^a^	*Φ*_em_	*k*_r_, s^−1^ ^b^	*k*_nr_, s^−1^ ^b^	*λ*_em_, nm	*τ*_av,_ µs^a^
**1W**	551	1.32	0.08	6.2 × 10^4^	6.9 × 10^5^	530	95
1W* (**1W** dried)^c^	461	11.82	0.73	6.2 × 10^4^	2.3 × 10^4^	481	233
**1M** (+MeOH vap.)	522	12.96	0.46	3.5×10^4^	4.2 × 10^4^	520	109
1M* (**1M** dried)^c^	525	6.01	—	—	—	—	—
**1E_b** (+EtOH vap.)	492	9.28	0.23	2.5 × 10^4^	8.3 × 10^4^	507	334
**1E_b** dried (1E*)^c^	525	2.71	0.09	3.3 × 10^4^	3.4 × 10^5^	516	212
**1E_y**	550	1.33	–^d^	—	—	487	270
**1DME** (crystals + DME vap.)	543	4.62	–^d^	—	—	535	138
**1DME^vap^** (1W* + DME vap.)	570	6.50	0.48	8.0×10^4^	8.0×10^4^	512	261

^a^ Amplitude‐weighted average emission lifetime is determined by the equation *τ_av_* = Σ*A_i_τ_i_*, *A_i_* = weight of the *i*th component.

^b^ *k_r_* = *Φ*_em_/*τ*_av_; *k* *_nrnr_* = (1 – *Φ*_em_)/*τ*_av._

^c^ Vacuum dried at 393 K for 10 h.

^d^ Could not be determined reliably due to the loss of solvent molecules.

The moderate blue luminescence of ethanol solvate **1E** **_** **b** (λ_em_ = 492 nm, *Φ*_em_ = 0.23) might be defined by short Na–I contacts, which widen the optical band gap by affecting the orbitals of the iodide to stabilize the ground state. The elongation of the observed lifetime from 9.28 µs (297 K) to 334 µs (80 K) corresponds to approximately 10‐fold decrease of *k*_r_ that, along with a small red shift to 507 nm, might suggest the TADF from the (M + X)LCT state (Figure S17, Table S3). The photoemission of the minor metastable form **1E** **_** **y** at ambient conditions peaks at visibly longer wavelength (*λ*_em_ = 550 nm) compared to both **1E** **_** **b** and **1M** (Figure 2 and Table 1). The intrinsic instability of **1E_y** toward intramolecular conversion to **1E** **_** **b** and loss of the solvent prevented accurate measurement of the quantum yield. The observed difference in the emission between two ethanol solvates **1E** **_** **y**/**1E** **_** **b** could be plausibly rationalized by the electronic and geometry alterations arising from the I–Na and Na–π bonding. For structurally analogous **1E** **_** **y** and **1M** we tentatively hypothesize that the Na–π contacts, which vary considerably between **1E** **_** **y** (*d*_Na–C min_ = 3.516 Å) and **1M** (*d*_Na–C min_ = 3.204 Å), implicitly influence the energies of the ground and excited states by imposing, for instance, tighter restrictions on the excited state structural relaxation in **1M**. The same argument could be applied to the emission of **1E** **_** **y** at 80 K, which, in contrast to **1M**, exhibits a large blue shift (550 → 487 nm, Figure 2 and Table 1) possibly due to frozen molecular flexibility and destabilization of ^3^CT states. Theoretical optimization of the ground state geometries of **1M** and **1E** **_** **y** and analysis of frontier molecular orbitals (Figures S15 and S18), however, did not show appreciable deviations in Na^…^C distances, energies, and orbital contributions of S_0_ → S_1_/S_2_ electronic transitions between these two forms (Table S3), which might be caused by intermolecular interactions and crystal packing not taken into account by the computational method.

The luminescence profile and observed lifetime for **1W**  virtually coincide with those for **1E** **_** **y**. Although the radiative rate of **1W** (6.2^.^10^4^ s^−1^) is almost twice as faster as that of **1M** (3.5^.^10^4^ s^−1^), the low quantum yield of the water solvate (*Φ*_em_ = 0.08) stems from dominating non‐radiative pathways (*k*_nr_ = 6.9^.^10^5^ s^−1^, Table 1). These relaxation channels could be provided by vibrational freedom of loosely bound water ligands and intramolecular conformational nonrigidity of the non‐symmetrical structure of **1W**.

Relatively weak binding of oxygen‐donor ligands prompted us to investigate the effect of their removal and substitution. Scheme 2 summarizes observed transformations. Vacuum drying of **1M**, **1E** **_** **b**, and **1W** at approximately 393 K (120°C) yields colorless desolvated materials 1M*, 1E*, and 1W*, respectively. Thermogravimetric analysis (TGA) of these samples shows decreases of weights to 0.942 (**1M**), 0.89 (**1E_b**), 0.949 (**1W**) which occur in the temperature range of 363–435 K (Figure S20) and can be assigned to the loss of four methanol (**1M**), four ethanol (**1E** **_** **b**), and five water (**1W** assuming it is a hexahydrate) molecules; the calculated residual fractions are 0.926, 0.897, and 0.947, respectively. In line with this hypothesis, the TGA of 1W* in the same temperature window shows only a minor weight loss to 0.987, which could be explained by its hygroscopic behavior. The elemental analysis indicates complete desolvation of the initial phases **1W** and **1E_b** (Table S4); that is, 1W* and 1E* are compatible with the formulation [CuI(***P^3^OO***)Na]_2_. 1M* is probably metastable and cannot be accurately analyzed due to its high hygroscopicity and nearly immediate phase change when opened to air.

**SCHEME 2:**
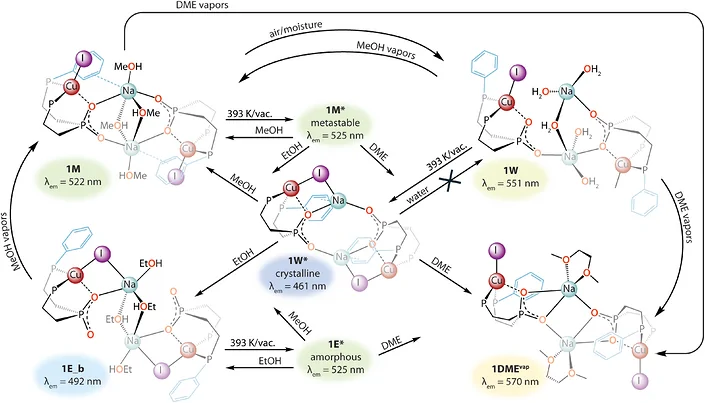
Summary of vapor and thermally induced interconversions between solvated and solvent‐free forms illustrating possible structural transformations.

1W*, which retains crystalline nature (see its PXRD, Figure S8), is the most blue‐shifted luminophore in the series (*λ*_em_ = 461 nm). The increased quantum yield of 0.73 under an inert atmosphere (Figure 2 and Table 1) and the lifetime of 11.82 µs deliver the radiative rate *K*_r_ = 6.2×10^4^ s^–^^1^ at room temperature, which is the same as that of the parent **1W**, whereas the nonradiative rate for 1W* is suppressed by 30‐fold to 2.3×10^4^ s^−1^. At 80 K, the emission peaks at *λ*_em_ = 481 nm, while the *k*_r_ does not exceed 4.2^.^10^3^ s^−1^, which is the value assuming unity *Φ*_em_. The order of magnitude slower *k*_r_ and a longer wavelength of luminescence at low temperature (Figure S13 and Table 1) testify in favor of the TADF mechanism operating for 1W* too. The removal of water is largely irreversible, and even prolonged treatment of the dried form 1W* with H_2_O vapors does not restore original crystalline **1W** but produces material with broad blue‐white luminescence likely resulting from a mixture of phases.

Drying the ethanol form **1E**_**b** yields amorphous solid 1E* (Figure S7), in contrast to 1W*. Weak luminescence of 1E* (*Φ*_em_ = 0.09, Figure S21) is seemingly caused by the loss of crystallinity and arguably the disruption of the molecular structure that indicates the importance of crystal packing in keeping the molecular integrity and attaining high quantum efficiency for these compounds [49, 50].

To get insight into the molecular changes driven by the desolvation–solvation, we performed DFT optimization of the ground state geometries of selected compounds. The predicted structure of **1M** satisfactorily agrees with the x‐ray diffraction data (Figure 3) and reproduces short C_Ph_^…^Na contacts and long I^…^Na separations, which are comparable to experimental values (Table S2). The removal of alcohol ligands from **1M**, **1E** **_** **y**, and **1E** **_** **b** leads to the structure of 1*, which is identical for the three parent systems. It features some shortening of C_Ph_^…^Na and significant contraction of I^…^Na distances by >1.3 Å (*d*_Na–I calc_ = 4.402/4.418 Å in **1M** and 3.037/3.064 Å in 1*, Figure 3) resulting in the formation of the I–Na bond, which is similar to that found in **1E** **_** **b** (*d*_Na–I_ = 3.176(1) Å). The lowest energy electronic transitions to the isoenergetic S_1_ and S_2_ states for the desolvated derivative 1* are of the same nature as those for **1M**, having (M+X)LCT (Cu‐I → π*) configurations (Figure 3, Figure S19, Table S3). Thus, despite the fact that sodium ions do not contribute to the frontier molecular orbitals and configurations of the lowest excited states, the contrasting emission change observed for 1W* versus **1W** and **1M** could be related to the contraction of I^…^Na connectivity and accompanying structural variations within the coordination sphere of the copper chromophore fragments, which are therefore indirectly regulated by the reversible binding of methanol molecules to the secondary‐sphere sodium ions. Nevertheless, as different phases are obtained upon solvent removal from **1M**, **1E_b**, and **1W**, crystal packing and molecular preorganization evidently play a major role in guiding solid‐state transformations among these species.

**FIGURE 3:**
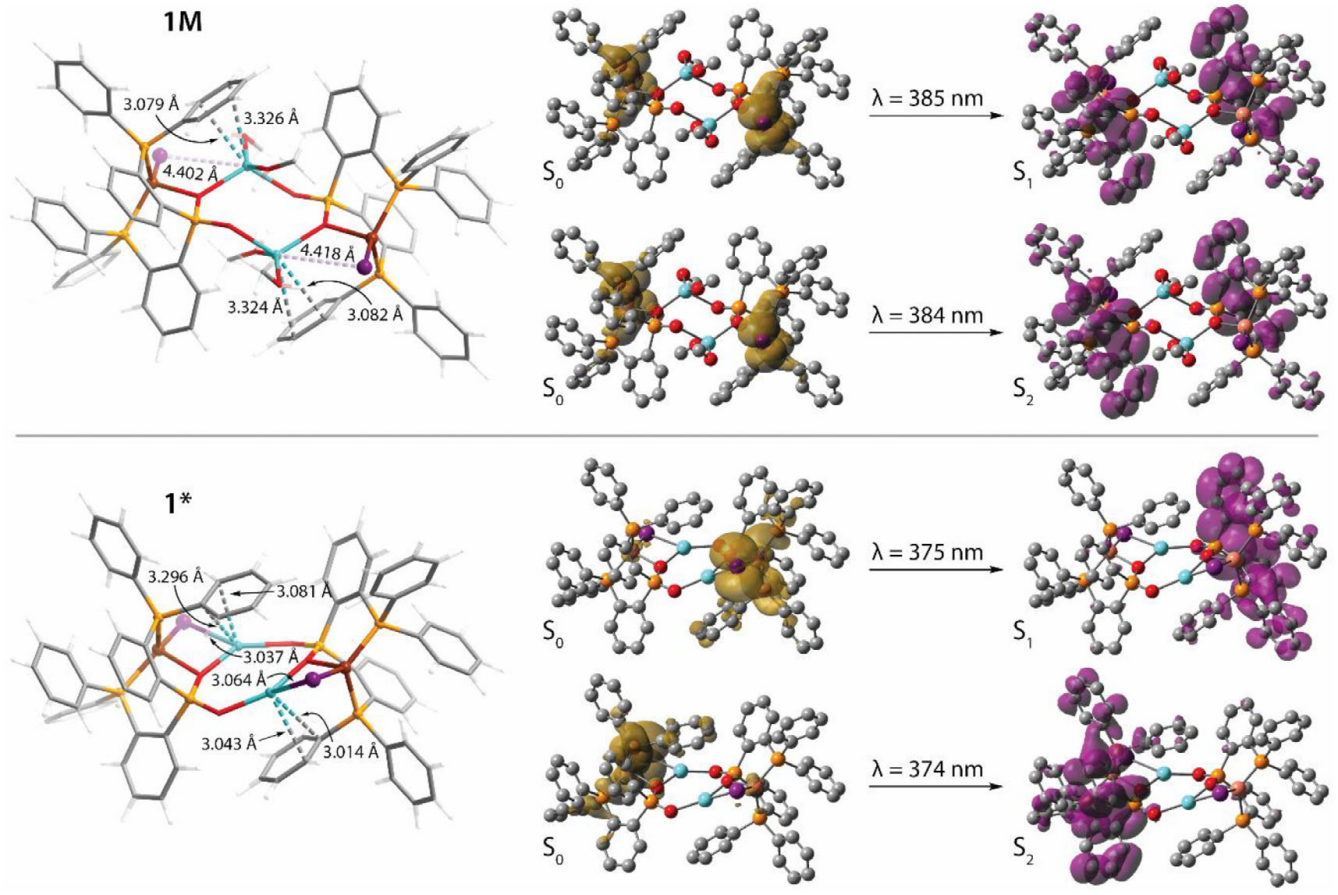
DFT‐optimized ground state (*S*_0_) geometries and electronic charge‐density difference plots for the vertical *S*_0_ → *S*_1_ and *S*_0_ → *S*_2_ transitions of compounds **1M** and 1* (for details, see the Supporting Information; mustard = density depletion, purple = density increase; 4.10^−4^ e^.^bohr^−3^).

All desolvated materials (1M*, 1E*, 1W*) are readily transformed into solvated forms (Scheme 2). The phases **1M**, **1W**, and **1E** **_** **b** are interconvertible through desolvation‐solvation cycles (drying at 393 K → exposure to vapors of alcohols/moisture), which occur within secondary coordination sphere of Na ions: 1M* (or 1W*) + EtOH vap. → **1E** **_** **b**, 1E* (or 1W*) + MeOH vap. → **1M**, and **1M** + moist air → **1W**. These transformations were confirmed by the PXRD analyses of the bulk phases (Figures S6–S10). The ability of solvent‐free materials 1W* and 1E* to generate phases **1M** and **1E** **_** **b** with clearly discernible photoemissions (522 and 492 nm) when treated with different alcohols illustrates a rare case of distinct responses to these two volatiles demonstrated by copper(I) emitters [26, 29, 51]. In a broader view, the cases of selective optical sensing of MeOH over EtOH remain rather limited mainly including some metal‐organic frameworks [52, 53] and inorganic [54] or hybrid materials [55, 56, 57].

Moreover, 1W* and 1E* demonstrate noticeable selectivity to MeOH when exposed to vapors of 1:1, 1:5, and 1:10 v/v mixtures of MeOH:EtOH for 60 min, resulting in the green‐emissive **1M** phase (Figure S10; **1M** formed from 1W* under 1:10 vapors converts into **1E** **_** **b** at prolonged exposure). The 1:100 v/v mixture of MeOH:EtOH transforms 1W*/1E* mainly into sky‐blue emissive **1E** **_** **b** modification. In addition, the transition 1W*/1E* → **1M** under fumes of a 1:1 v/v MeOH:pyridine mixture highlights the Na‐defined preference of the mixed Cu–Na system to bind some oxygen donors, which is otherwise unusual for Cu(I) luminescent compounds mostly comprising softer N‐, P‐donor ligands. Nonetheless, it is worth emphasizing that the coordination of alcohols is evidently enhanced by intramolecular ROH^…^O/I hydrogen bonding. The non‐chelating ethers (diethyl ether, tetrahydrofuran, and dioxane) or acetone lacking hydroxide groups do not form analogous isolable solvates.

Further utilizing the adaptable coordination environment of sodium, the exposure of the solvent‐free samples 1W* or 1E* to chelating 1,2‐dimethoxyethane (DME) produces microcrystalline material **1DME^vap^** as confirmed by the PXRD data (Figure S9). This phase, however, differs from the structurally characterized **1DME** that is also manifested by their optical properties. Based on the TGA analysis (Figure S20), both forms lose approximately 10% of the initial weight at temperatures above 475 K, which correlates well with 10.1% mass fraction corresponding to two DME ligands. Neat crystals of **1DME** are moderately luminescent in a yellow‐greenish region peaking at 543 nm at room temperature (Table 1, Figure S22), likely pointing to the TADF due to approximately 30‐fold increase of the lifetime at 80 K. The **1DME^vap^** phase exhibits bright yellow emission (*λ*_em_ = 570 nm, *Φ*_em_ = 0.48, Figure 2), which turns green upon decreasing the temperature (*λ*_em_ = 512 nm at 80 K). Although structural details of **1DME^vap^** are not available, it is reasonable to anticipate the lack of close Na–π and Na–I contacts as in **1DME** (Figure S5) due to comparable steric restraints dictated by the bulky DME ligands. The resemblance of long wavelength emissions at 297 K and pronounced blue shifts at 80 K demonstrated by **1E_y** and **1DME^vap^** apparently imply that akin effects related to Na–π interactions and structural non‐rigidity might affect the relaxation of the excited states at different temperatures.

Based on the results presented above, we are currently extending this coordination strategy to a broader family of alkali metal halides with systematic variation of ***P^3^OO* ^–^**:Cu:M:Hal stoichiometry (M = Li, K, Cs, Rb; Hal = Cl, Br, I) and crystallization conditions. These studies aim to establish structure–property relationships linking aggregate topology to their photophysical behavior.

## 3 Conclusion

The reaction of a hybrid phosphane‐(sodium phosphinate) [***P* ^*3*^ *OO***]Na with copper(I) iodide leads to the assembly of two anionic {CuI(***P^3^OO***)}^–^ units around Na^+^ ions. The coordination of oxygen‐containing solvents as ligands to sodium centers gives methanol (**1M**), ethanol (major **1E** **_** **b** and minor **1E** **_** **y**), water (**1W**), and dimethoxyethane (**1DME**) solvated forms. These materials show intense sky‐blue to yellow photoluminescence that is consistent with the TADF and involves the (M + X)LCT (Cu,I → aryl) configuration of the excited state. Although sodium ions do not contribute to the lowest energy electronic transitions, the perturbation of their coordination environments affects the copper‐phosphane chromophores and substantially alters the photophysical behavior of titled compounds. The removal of weakly bound water from weakly yellow‐emissive **1W** affords solvent‐free modification 1W* showing the most blue‐shifted emission (461 nm) and the highest quantum yield (0.73) within the studied series. The desolvated material 1W* is readily converted into solvates **1M**, **1E** **_** **b**, and **1DME^vap^** upon exposure to corresponding organic vapors that results in a high‐contrast change of the emission with red shifts up to >100 nm. Theoretical analysis suggested that a significant contraction of the Na^…^I distances accompany the loss of the solvent and likely induce blue luminescence of 1W*, whereas the longer‐wavelength yellow emissions of **1E** **_** **y**, **1W**, and **1DME^vap^** are tentatively ascribed to loosened Na–I/π connectivities and molecular flexibility. Remarkably, these distinct optical responses allow for the discrimination of methanol and ethanol vapors as well as for the detection of methanol in up to 1:10 methanol:ethanol mixtures.

These results demonstrate that secondary‐sphere interactions involving reversible coordination of oxygen donors to optically silent alkali metal ions can be a very efficient tool for modulating photoluminescence of the copper(I)‐sodium aggregate. We anticipate that the construction of heterometallic d‐s or d‐p assemblies from the mixed soft‐hard donor ligands represents a new perspective for stimuli‐responsive materials, in which the main group element performs a specific function (e.g., guest binding/releasing) and triggers photophysical changes of the transition metal chromophore.

## Author Contributions

**Henna Korhonen**: investigation, methodology, conceptualization, writing – review and editing. **Dorota Głosz**: investigation, methodology. **Mariia Beliaeva**: conceptualization, investigation, methodology. **Andrey Belyaev**: conceptualization, investigation, data curation, supervision, validation, methodology. **Constance Lecourt**: investigation, methodology. **Adrien Schlachter**: investigation, methodology. **Karine Costuas**: investigation, methodology, data curation, writing – review and editing. **Guillaume Calvez**: investigation, validation, data curation. **Robert Podgajny**: conceptualization, supervision. **Christophe Lescop**: conceptualization, supervision, writing – review and editing, funding acquisition, validation. **Igor O. Koshevoy**: data curation, supervision, funding acquisition, writing – original draft, writing – review and editing, validation, project administration.

## Conflicts of Interest

The authors declare no conflicts of interest.

## Supporting information



Experimental procedures, additional spectroscopic, crystallographic, and computational (XYZ) data. The authors have cited additional references within the Supporting Information [58, 59, 60, 61, 62, 63, 64, 65, 66, 67, 68, 69, 70, 71, 72, 73, 74, 75].**Supporting File 1**: anie74773‐sup‐0001‐SuppMat.docx.

**Supporting File 2**: anie74773‐sup‐0002‐SuppMat.rar.

## Data Availability

The data that support the findings of this study are openly available in CCDC at https://www.ccdc.cam.ac.uk/structures/, reference numbers 2549124, 2549125, 2549126, 2549127, and 2582510.
