# Supplementary material for: Luminescence Vapochromism of a Cu(I)/Na Phosphane Iodide Assembly Based on a Secondary‐Sphere Sodium Response
Source: Angew Chem Int Ed Engl. 2026 Sep 15;65(44):e6117838. doi: 10.1002/anie.6117838 (PMC13703896; doi:10.1002/anie.6117838)
Supplement: Supplementary file 1 [file ANIE-65-e6117838-s001.docx]

*Supporting information for*

*Luminescence Vapochromism of a Cu(I)/Na Phosphane Iodide Assembly Based on a Secondary-Sphere Sodium Response*

*Henna Korhonen,^a^ Dorota Głosz,^b^ Mariia Beliaeva,^a^ Andrey Belyaev,^a^* Constance Lecourt,^c^* *Adrien Schlachter,^c^ Karine Costuas,^c^* Guillaume Calvez,^c^ Robert Podgajny,^b^ Christophe Lescop,^c^* Igor O. Koshevoy^a^**

*^a^ Department of Chemistry and Sustainable Technology, University of Eastern Finland, Yliopistokatu 7, Joensuu, 80101, Finland*

*^b^ Faculty of Chemistry, Jagiellonian University in Krakow, Gronostajowa 2, 30-387 Kraków, Poland*

*^c^ University of Rennes, INSA Rennes, CNRS, ISCR (Institut des Sciences Chimiques de Rennes), UMR 6226, F-35000 Rennes, France*

E-mail: [andrei.beliaev@uef.fi](mailto:Andrei.beliaev@uef.fi); [karine.costuas@univ-rennes1.fr](mailto:karine.costuas@univ-rennes1.fr); [christophe.lescop@insa-rennes.fr](mailto:christophe.lescop@insa-rennes.fr); [igor.koshevoy@uef.fi](mailto:igor.koshevoy@uef.fi)

**Table of Contents**

| ***Experimental section*** | S3 |
| --- | --- |
| ***Figure S1***. ^1^H and ^31^P{^1^H} NMR spectra of **1M**. | S3 |
| ***Figure S2***. Fully optimized structure of **1E_b**. | S6 |
| ***Table S1***. Crystal data and structure refinement for **1M**, **1E_b**, **1E_y**, **1W** and **1DME**. | S7 |
| ***Table S2***. Selected bond lengths and angles for **1M**, **1E_b**, **1E_y**, **1W** and **1DME**. | S9 |
| ***Figures S3****–****S5***. Molecular views of **1E_y**, **1DME** and **1W**. | S10-S11 |
| ***Figures S6****–****S10***. Powder XRD patterns of **1M**, **1E_b**, **1E_y**, **1W**, **1W***, **1DME**. | S11–S14 |
| ***Figure S11***. Time-dependent emission spectra of **1M** exposed to ambient air (297 K). | S15 |
| ***Figure S12***. Photoluminescence intensity decays for **1M/1M***, **1E_b/1E***, **1W/1W***, **1DME**, **1DME^vap^** and [***P*^3^*OO***]Na in the solid state. | S16 |
| ***Figure S13***. VT Excitation and emission spectra of solid compounds at 80 K. | S17 |
| ***Figure S14***. Excitation and emission spectra of [***P*^3^*OO***]Na in the solid state. | S18 |
| ***Table S3***. Energy, wavelength, oscillator strength and nature of the three first singlet vertical electronic excitations for the ligand [***P*^3^*OO***]Na and complexes **1***, **1M**, **1E_y**, **1E_b**. | S19 |
| ***Figures S15–S19***. Isosurface representations of the frontier molecular orbitals and the electronic charge-density difference between the first vertical excited states and the ground state of [***P^3^OO***]Na, **1M**, **1***, **1E_b**, **1E_y**. | S21–S25 |
| ***Table S4***. Analytical data for samples of **1M**, **1E_b** and their dried derivatives. | S26 |
| ***Figure S20***. Thermogravimetric analyses (TGA) of **1M**, **1W**, **1W***, **1E_b**, **1DME** and **1DME^vap^**. | S26 |
| ***Figure S21***. Excitation and emission spectra of compound **1E*** at 297 and 80 K. | S27 |
| ***Figure S22***. Excitation and emission spectra of crystalline **1DME** at 297 and 80 K. | S27 |
| ***References*** | S28 |
|  |  |
|  |  |
|  |  |
|  |  |

**Experimental section**

**General comments.** Sodium bis(2-(diphenylphosphaneyl)phenyl)phosphinate [***P*^3^*OO***]Na was prepared according to the published procedure.^[1]^ Other reagents and solvents were used as received. CHN Microanalyses were carried out on an Elementar Vario Micro Cube instrument at the analytical laboratory of the University of Eastern Finland.

**Synthesis and crystallization**. **[CuI(*P^3^OO*)Na(MeOH)_2_]_2_ (1M)**. [***P***^3^***OO***]Na (50 mg, 0.08 mmol), CuI (16 mg, 0.08 mmol) and NaI (24 mg, 0.16 mmol) were dissolved in methanol (10 mL), and the solution was stirred under a nitrogen atmosphere for 15 minutes. The solvent was evaporated *in vacuo*. Pale residue was recrystallized by a gas-phase diffusion of diethyl ether into a methanol/toluene solution of crude **1M** at 4 ⁰C to give colorless block crystals, which were washed with methanol, diethyl ether and briefly dried on air (52 mg, 73 %). Identical crystalline material can be obtained by a slow evaporation of a methanol/water solution at room temperature in the presence of an excess of NaI. ^1^H NMR (500 MHz, CD_2_Cl_2_ + 5% MeOD-*d*_4_, 293 K; *δ*): 8.05 (t, *J*_HH_ 9.5 Hz, 2H), 7.60 (s br, 4H), 7.32 (s br, 8H), 7.18 (t, *J*_HH_ 7.5 Hz, 2H), 7.07 (t, *J*_HH_ 7.6 Hz, 6H), 6.93 (s br, 4H), 6.69 (s br, 2H). ^31^P{^1^H} NMR (202 MHz, CD_2_Cl_2_ + 5% MeOD-*d*_4_, 293 K; *δ*): 21.6 (s, 1P), -12.9 (s br, 2P). Anal. Calcd for C_76_H_72_Cu_2_I_2_Na_2_O_8_P_6_: C, 52.88; H, 4.20. Found: C, 52.65; H, 4.17.

***Figure S1***. ^1^H and ^31^P{^1^H} (NMR spectra of **1M** (CD_2_Cl_2_ + 5% MeOD-*d*_4_, 293 K).

**[CuI(*P^3^OO*)Na(H_2_O)_x_]_2_ (1W)**. Crystals of **1M** were kept under ambient conditions for 15 h. The resulting material retained crystallinity and complete exchange of methanol for water molecules was confirmed by single crystal and PXRD analyses. **1W** is not formed via crystallization as slow evaporation of a methanol-water solution in the presence of NaI yields exclusively **1M** that indicates the preference for methanol coordination.. Anal. Calcd for C_72_H_56_Cu_2_I_2_Na_2_O_4_P_6_^.^6H_2_O: C, 50.69; H, 4.02. Found: C, 50.50; H, 4.06.

**[CuI(*P^3^OO*)Na(EtOH)_2_]_2_ (1E_b/1E_y)**. Prepared analogously to **1M** using ethanol instead of methanol. Recrystallization at room temperature gave colorless block crystals of the major blue luminescent form **1E_b** and a few block crystals of metastable minor modification showing yellow luminescence **1E_y** (70 %). **1E_y** spontaneously transforms into **1E_b** upon prolong standing in mother liquor. Anal. Calcd for C_80_H_80_Cu_2_I_2_Na_2_O_8_P_6_: C, 53.91; H, 4.52. Found: C, 53.65; H, 4.78.

**[CuI(*P^3^OO*)Na(MeOCH_2_CH_2_OMe)_2_]_2_ (1DME)**. A solvent-free form of complex **1** (20 mg) was dissolved in dichloromethane (2 ml) and 1,2-dimethoxyethane (DME, 0.2 ml) was added. Slow evaporation of this solution at room temperature gave colorless block crystals of **1DME** with yellowish luminescence (76 %). Drying the sample results in a rapid loss of the DME ligand and therefore prevented accurate elemental analysis.

**X-ray Structure Determination.** The crystals of **1M**, **1E_b**, **1E_y**, **1W** and **1DME** were immersed in cryo oil, mounted in a Nylon loop, and measured at a temperature of 150 K. The diffraction data were collected with Bruker Kappa Apex II, Rigaku SuperNova and Rigaku Synergy S diffractometers using Mo K*α* (λ = 0.71073 Å) and Cu K*α* (*λ* = 1.54184 Å) radiations. The APEX2^[2]^ and CrysAlisPro^[3]^ program packages were used for cell refinements and data reductions. A numerical absorption correction (SADABS^[4]^ or CrysAlisPro^[3]^) was applied to all data. The structures were solved by direct methods using the SHELXT-2018^[5]^ program with the WinGX^[6]^ and Olex2^[7]^ graphical user interfaces. Structural refinements were carried out using SHELXL-2019.^[5]^

Non-coordinated crystallization solvent in **1E_b** could not be resolved unambiguously. The contribution of the missing solvent to the calculated structure factors was taken into account by using a *SQUEEZE* routine of *PLATON*.^[8]^ The missing solvent was not taken into account in the unit cell content. The DME ligand and the Na(1) atom were modeled to be disordered between two positions with occupancies of 0.71/0.29. A series of geometry and displacement constraints and restraints were applied to these moieties.

The O(4)–H hydrogen atom in **1E_y** and water H atoms in **1W** were located from the difference Fourier map and constrained to ride on their parent atoms with *U*_iso_ = 1.5 *U*_eq_ (parent atom). Other H atoms were positioned geometrically and constrained to ride on their parent atoms C–H = 0.95–0.99 Å, O–H = 0.84 Å, and *U*_iso_ = 1.2–1.5 *U*_eq_ (parent atom). The crystallographic details are summarized in Table S1.

**Powder X-ray Diffraction Analysis (PXRD):** Data were collected from bulk polycrystalline material (three averaged replicas from ground crystalline material) using a Rigaku XtaLAB Synergy-S with Cu K_α_ radiation (λ = 1.54184 Å, Microfocus beam, Divergence 2.3, 600 sec exposure time) with Gandolfi strategy for data collection. Calculated data were generated using Mercury 2024.3.1 (Build 428092) software.

**Photophysical Measurements.** Both excitation and emission spectra of the samples were recorded on Edinburgh Instrument FLS1000 and Horiba Jobin-Yvon Fluorolog-3 spectrometers. The quantum yields in the solid-state at room temperature were measured on the FLS1000 and Fluorolog-3 equipped with an integrating sphere. For the sake of accuracy, they have been measured three times, and the value given is obtained from the averaging of these measurements. The excitation and emission spectra were corrected using the standard corrections supplied by the manufacturer for the spectral power of the excitation source and the sensitivity of the detector.

The luminescence lifetimes were measured using a pulsed Xenon microsecond flashlamp or a pulsed laser with multichannel scaling (MCS) or Time-Correlated Single Photon Counting (TCSPC) modules. Steady-state and time-resolved low temperature measurements were performed utilizing an Oxford Optistat DN cryostate. Solid sample was placed in a quartz sample holders inside the integrating sphere and the cryostat and maintained at the desired temperature until equilibrium was reached before recording the spectrum.

**DFT calculations.** Density functional Theory (DFT) calculations of the listed systems were carried out with the ORCA 5.0.4 program.^[9]^ The PBE0^[10-11]^ functional was employed associated with the SVP^[12-13]^ atomic basis set. D3BJ dispersion correction was used.^[14]^ DFT for S_0_ and Tamm Dancoff approximation time-dependent DFT (TDA-DFT) were employed.^[15-17]^ Tight criteria of energy convergence were applied to ensure high quality of the results (10^−6^ u.a.). Vibrational frequency calculations were performed only for the ligand study due to computational limitations. Full geometry optimizations of the ground states were performed for all cases except for compound **1E_b**. In the latter case, one intramolecular π–π stacking and one sodium-π interactions^[18]^ occurs upon full geometry optimization of the molecular XRD determined structure (Figure ST1).

***Figure S2***. Fully optimized structure of **1E_b**. Carbon atoms involved in intramolecular π–π stacking and in the sodium-π interactions are represented in light pink. Circular inset at bottom left: other view of the π–π stacking. Circular inset at bottom right: other view of the sodium-π interaction.

These intramolecular interactions are inoperant in the solid state due to energetic prevalence of intermolecular contacts. In order to have some relevant information on the electronic structure of **1E_b** and its electronic excitation properties, constrained geometry optimization was performed (two distances fixed to their crystallographic value: between the C_ipso_ and the closest C of the second cycle of the phenyl ring involved in the stacking, and between the two hydrogen atoms which distance is the more shortened upon creation of the sodium-π interactions upon full geometry optimization). The energy, oscillator strength, and nature of the lowest electronic singlet and triplet excited states (S_1_, S_2_, S_3_, T_1_, T_2_, T_3_) are given in Table S3. The frontier molecular orbitals (MO) involved in the transitions that describe these excited states, i.e. highest occupied molecular orbitals (HOMOs) and the lowest unoccupied molecular orbitals (LUMOs), the electronic charge-density differences between the first vertical singlet excited states (S_1_ and S_2_) and the ground state (S_0_) are represented in Figures 3, S15–S19. The iso-surface representations were done using the GaussView program.^[19]^ Hydrogen atoms are omitted in most of the representations for sake of clarity. The colors of atoms in the structural representations are: sodium = light blue, carbon = gray, oxygen = red, phosphorus = orange, iodine = purple, copper = salmon.

***Table S1***. Crystal data and structure refinement for **1M**, **1E_b**, **1E_y**, **1W** and **1DME**.

|  | | **1M** | **1E_b** | **1E_y** | **1W** | **1DME** |
| --- | --- | --- | --- | --- | --- | --- |
| CCDC | | 2549124 | 2549125 | 2549126 | 2582510 | 2549127 |
| Empirical formula | | C_76_H_72_Cu_2_I_2_Na_2_O_8_P_6_ | C_80_H_80_Cu_2_I_2_Na_2_O_8_P_6_ | C_80_H_80_Cu_2_I_2_Na_2_O_8_P_6_ | C_72_H_68_Cu_2_I_2_Na_2_O_10_P_6_ | C_80_H_76_Cu_2_I_2_Na_2_O_8_P_6_ |
| Formula weight | | 1726.01 | 1782.12 | 1782.12 | 1705.94 | 1778.08 |
| Temperature (K) | | 150(2) | | | | |
| Wavelength (Å) | | 0.71073 | | | | 1.54184 |
| Crystal system | | Triclinic | Monoclinic | Triclinic | Triclinic | Triclinic |
| Space group | | *P*$\bar{1}$ | *C* 2/*c* | *P*$\bar{1}$ | *P*$\bar{1}$ | *P*$\bar{1}$ |
| Unit cell dimensions | |  |  |  |  |  |
| a (Å) | | 10.4961(5) | 32.454(2) | 10.6320(4) | 12.1808(3) | 10.5109(2) |
| b (Å) | | 11.0220(5) | 12.9312(8) | 11.9846(5) | 17.6948(5) | 12.1211(2) |
| c (Å) | | 17.6866(11) | 22.6192(18) | 15.7434(6) | 18.6994(8) | 16.7320(2) |
| a (^o^) | | 100.533(3) | 90 | 81.211(3) | 63.416(3) | 90.910(2) |
| β (^o^) | | 100.503(3) | 117.550(3) | 71.531(3) | 89.305(2) | 98.463(2) |
| γ (^o^) | | 110.874(2) | 90 | 83.731(3) | 87.474(2) | 110.832(2) |
| Volume (Å^3^) | | 1810.16(17) | 8416.3(11) | 1876.41(13) | 3600.7(2) | 1965.29(6) |
| Z | | 1 | 4 | 1 | 2 | 1 |
| ρ_calc_ (Mg/m^3^) | | 1.583 | 1.406 | 1.577 | 1.573 | 1.502 |
| μ (mm^-1^) | | 1.640 | 1.414 | 1.585 | 1.650 | 8.527 |
| F(000) | | 868 | 3600 | 900 | 1712 | 896 |
| Crystal size (mm^3^) | | 0.486×0.433×0.342 | 0.568×0.522×0.438 | 0.350×0.270×0.220 | 0.253×0.146×0.097 | 0.084×0.057×0.016 |
| θ range for data collection (^o^) | | 2.054 to 38.283 | 2.163 to 27.000 | 2.335 to 32.101 | 2.132 to 30.420 | 2.677 to 79.761 |
| Index ranges | | -15≤h≤17,  -19≤k≤17,  -29≤l≤28 | -41≤h≤40,  -16≤k≤16,  -28≤l≤28 | -15≤h≤14,  -15≤k≤17,  -23≤l≤23 | -17≤h≤16,  -21≤k≤24,  -17≤l≤25 | -11≤h≤13,  -15≤k≤15,  -21≤l≤21 |
| Reflections collected | | 52911 | 59037 | 25545 | 35427 | 31173 |
| Independent reflections | | 17239 | 9184 | 11853 | 19115 | 8286 |
| R_int_ | | 0.0183 | 0.0192 | 0.0355 | 0.0211 | 0.0516 |
| Completeness to θ = 25.24^o^ (Mo)/67.684°(Cu) | | 99.9 % | 99.8 % | 99.9 % | 99.9 % | 100 % |
| Absorption correction | | Numerical | | | | |
| Max. and min. transmission | | 0.604 and 0.503 | 0.538 and 0.452 | 1.000 and 0.458 | 1.000 and 0.730 | 0.927 and 0.610 |
| Data/ restraints/ parameters | | 17239 / 0 / 437 | 9184 / 0 / 451 | 11853 / 0 / 451 | 19115 / 0 / 855 | 8286 / 25 / 515 |
| GOOF on F^2^ | | 1.027 | 1.034 | 1.047 | 1.054 | 1.043 |
| Final R indices [I>2σ(I)]^a^ | | R_1_ = 0.0220, wR_2_ = 0.0548 | R_1_ = 0.0252, wR_2_ = 0.0600 | R_1_ = 0.0345, wR_2_ = 0.0631 | R_1_ = 0.0438, wR_2_ = 0.0840 | R_1_ = 0.0492, wR_2_ = 0.1192 |
| R indices (all data) | | R_1_ = 0.0264, wR_2_ = 0.0566 | R_1_ = 0.0287, wR_2_ = 0.0625 | R_1_ = 0.0483, wR_2_ = 0.0720 | R_1_ = 0.0658, wR_2_ = 0.0941 | R_1_ = 0.0556, wR_2_ = 0.1229 |
| Largest diff. peak and hole (e.Å^-3^) | | 1.145 and -0.895 | 2.230 and -1.224 | 0.778 and -1.241 | 1.293 and -1.361 | 1.298 and -1.333 |
|  | ^a^ R_1_ = Σ\|\|F_o_\| – \|F_c_\|\|/Σ\|F_o_\|; wR_2_ = [Σ [w(F_o_^2^ – Fc^2^)^2^]/ Σ[w(F_o_^2^)^2^]]^1/2^ | | | | | |

***Table S2***. Selected bond lengths (Å) and angles (^o^) for **1M**, **1E_b**, **1E_y**, **1W** and **1DME**.

|  | **1M** | **1E_b** | **1E_y** | **1W*^a^*** | **1DME** |
| --- | --- | --- | --- | --- | --- |
|  | **Bond lengths, Å** | | | | |
| O(1)-Cu(1) | 2.2141(7) | 2.146(1) | 2.231(1) | 2.266(2)  2.201(2) | 2.343(3) |
| P(1)-Cu(1) | 2.2390(3) | 2.2434(6) | 2.2491(6) | 2.2421(8)  2.2414(8) | 2.2427(10) |
| P(2)-Cu(1) | 2.2645(3) | 2.2262(5) | 2.2703(5) | 2.2591(7)  2.2699(7) | 2.2671(10) |
| Cu(1)-I(1) | 2.5572(2) | 2.5833(3) | 2.5535(3) | 2.5510(4) | 2.5525(6) |
| O(1)-Na(1) | 2.3337(8) | 2.278(2) | 2.354(2) | 2.399(2)  3.806(2) | 2.480(11)*^b^* |
| O(2)-Na(1´/2) | 2.5372(9) | 4.011(2) | 2.500(2) | 2.223(3)  2.384(2) | 2.185(12) |
| Na(1)^…^I(1) | 4.6981(6) | 3.176(1) | 4.7212(9) | 4.789(1)  4.026(2) | 6.14(1)*^a^* |
| O(4/6)^…^I(1) | 3.493(1) | – | 3.592(2) | 3.624(3) | – |
|  | **Bond angles, ^o^** | | | | |
| P(1)-Cu(1)-P(2) | 118.49(1) | 118.06(2) | 115.35(2) | 118.78(3)  119.15(3) | 116.64(4) |
| O(1)-Cu(1)-P(1) | 95.94(2) | 88.29(4) | 95.87(4) | 95.43(5)  94.88(5) | 92.21(7) |
| O(1)-Cu(1)-P(2) | 79.58(2) | 97.75(4) | 79.75(4) | 80.28(5)  80.31(5) | 76.96(7) |
| P(1)-Cu(1)-I(1) | 119.487(9) | 116.50(2) | 120.60(2) | 118.69(2)  124.47(2) | 122.52(3) |
| P(2)-Cu(1)-I(1) | 115.132(9) | 122.89(2) | 115.03(2) | 116.42(2)  113.11(2) | 112.48(3) |
| O(1)-Cu(1)-I(1) | 119.69(2) | 99.43(4) | 112.20(3) | 118.35(5)  111.47(5) | 127.03(7) |
| Cu(1)-O(1)-Na(1) | 121.08(3) | 104.96(6) | 118.63(6) | 121.10(9) | 152.3(3)*^b^* |

*^a^* two values correspond to non-centrosymmetric molecule; *^b^* the values correspond to the main component of the disordered motif.

***Figure S3***. Molecular view of the metastable ethanol solvate **1E_y** (displacement ellipsoids are at 50% probability level).

***Figure S4***. Molecular views of the 1,2-dimethoxyethane solvate **1DME** (displacement ellipsoids are at 50% probability level; the DME ligand and Na atom are disordered, minor component with the occupancy of 0.29 is shown in semi-transparent mode). The right view in ball-and-stick highlights weak Na–π interaction (two C^…^Na distances correspond to the shortest separations for main/minor disordered components).

***Figure S5***. Molecular view of the water solvate **1W** (displacement ellipsoids are at 50% probability level).

***Figure S6***. Simulated and experimental powder XRD patterns of **1M** under different conditions (297 K).

***Figure S7***. Simulated and experimental powder XRD patterns of **1E_b** and **1E_y** under different conditions (297 K).

***Figure S8***. Simulated and experimental powder XRD patterns of **1W** under different conditions (297 K, simulated pattern for **1M** is shown for comparison).

***Figure S9***. Simulated and experimental powder XRD patterns of **1DME** under different conditions (297 K).

***Figure S10***. Powder XRD patterns of **1W*** (A, B) and **1E*** (A, B) exposed to vapors of solvent mixtures for 60 min. (297 K); (C) normalized emission spectra **1E*** corresponding to (B).

***Figure S11***. Normalized time-dependent emission spectra of **1M** exposed to ambient air (297 K).

***Figure S12***. Photoluminescence intensity decays for **1M**, **1E_b/1E***, **1W/1W***, **1DME**, **1DME^vap^** and [***P*^3^*OO***]Na in the solid state at 297 and 80 K.

***Figure S13***. (A) normalized excitation (left) and emission (right) spectra of solid compounds at 80 K; (B) VT emission spectra and observed lifetimes for **1M**; (C) VT emission spectra and observed lifetimes for **1W***.

***Figure S14***. Normalized excitation (dashed lines) and emission (solid lines) spectra of [***P*^3^*OO***]Na in the solid state at 297 and 80 K.

***Table S3***. Energy, wavelength, oscillator strength and nature in terms of combination of transitions between molecular orbitals (>3 %) of the three first singlet (S_0_➝S_n_) vertical electronic excitations for the ligand [***P*^3^*OO***]Na and complexes **1***, **1M**, **1E_y**, **1E_b**.

| **Compound** | **S_0_➝S_n_** | **Energy, eV (wavelength, nm)** | ***f*** | **Nature** |
| --- | --- | --- | --- | --- |
| [***P*^3^*OO***]Na | S_0_ ➝ S_1_ | 3.802  (326) | 0.048 | HOMO➝LUMO (51 %)  HOMO-1➝LUMO (46%) |
|  | S_0_ ➝ S_2_ | 3.936  (315) | 0.002 | HOMO➝LUMO+1 (51 %)  HOMO-1➝LUMO+1 (47 %) |
|  | S_0_ ➝ S_3_ | 4.054  (306) | 0.17 | HOMO-1➝LUMO (36 %)  HOMO➝LUMO (26 %)  HOMO➝LUMO+3 (24 %)  HOMO➝LUMO+2 (9 %)  HOMO-1➝LUMO+1 (4 %) |
| **1*** | S_0_ ➝ S_1_ | 3.303  (375) | 0.016 | HOMO-1➝LUMO (91 %)  HOMO➝LUMO+1 (4 %) |
|  | S_0_ ➝ S_2_ | 3.316  (374) | 0.040 | HOMO➝LUMO+1 (88 %)  HOMO-1➝LUMO (4 %) |
|  | S_0_ ➝ S_3_ | 3.457  (359) | 0.002 | HOMO➝LUMO+3 (66 %)  HOMO-1➝LUMO+2 (18 %)  HOMO➝LUMO+2 (5 %)  HOMO➝LUMO (3 %) |
| **1M** | S_0_ ➝ S_1_ | 3.223  (385) | 0.000 | HOMO➝LUMO+1 (52 %)  HOMO-1➝LUMO (42 %) |
|  | S_0_ ➝ S_2_ | 3.228  (384) | 0.059 | HOMO-1➝LUMO (48 %)  HOMO➝LUMO+1 (38 %)  HOMO➝LUMO (5 %) |
|  | S_0_ ➝ S_3_ | 3.380  (367) | 0.003 | HOMO➝LUMO+1 (39 %)  HOMO➝LUMO+2 (28 %)  HOMO-1➝LUMO+3 (13 %)  HOMO-2➝LUMO+1 (10 %) |
| **1E_y** | S_0_ ➝ S_1_ | 3.219  (385) | 0.000 | HOMO➝LUMO+1 (28 %)  HOMO➝LUMO (25 %)  HOMO-1➝LUMO (22 %)  HOMO-1➝LUMO+1 (22 %) |
|  | S_0_ ➝ S_2_ | 3.224  (385) | 0.064 | HOMO-1➝LUMO (50 %)  HOMO➝LUMO+1 (43 %) |
|  | S_0_ ➝ S_3_ | 3.404  (364) | 0.001 | HOMO-1➝LUMO+2 (45 %)  HOMO➝LUMO+3 (28 %)  HOMO-1➝LUMO+3 (11 %)  HOMO➝LUMO+2 (3 %) |
| **1E_b** | S_0_ ➝ S_1_ | 3.088  (401) | 0.009 | HOMO➝LUMO+1 (95 %) |
|  | S_0_ ➝ S_2_ | 3.100  (400) | 0.006 | HOMO-1➝LUMO (96 %) |
|  | S_0_ ➝ S_3_ | 3.350  (370) | 0.005 | HOMO-2➝LUMO+1 (94 %)  HOMO➝LUMO+3 (4 %) |

***Figure S15***. Isosurface representations of the frontier molecular orbitals HOMO-1 to LUMO+1 (± 2.10^-2^ (e.bohr^-3^)^½^), and the electronic charge-density difference between the first vertical singlet excited states (S_1_, S_2_) and the ground state (S_0_) of [***P^3^OO***]Na (mustard = density depletion, purple = density increase; 4.10^-4^ e.bohr^-3^).

***Figure S16***. Isosurface representations of the frontier molecular orbitals HOMO-1 to LUMO+1 (± 2.10^-2^ (e.bohr^-3^)^½^), and of the electronic charge-density difference between the first singlet (S_1_, S_2_) or triplet (T_1_, T_2_) vertical excited states and the ground state (S_0_) of **1M** (mustard = density depletion, purple = density increase; 4.10^-4^ e.bohr^-3^).

***Figure S17***. Isosurface representations of the frontier molecular orbitals HOMO-1 to LUMO+1 (± 2.10^-2^ (e.bohr^-3^)^½^), and of the electronic charge-density difference between the first singlet (S_1_, S_2_) or triplet (T_1_, T_2_) vertical excited states and the ground state (S_0_) of **1E_b** (mustard = density depletion, purple = density increase; 4.10^-4^ e.bohr^-3^).

***Figure S18***. Isosurface representations of the frontier molecular orbitals HOMO-1 to LUMO+1 (± 2.10^-2^ (e.bohr^-3^)^½^), and of the electronic charge-density difference between the first singlet (S_1_, S_2_) or triplet (T_1_, T_2_) vertical excited states and the ground state (S_0_) of **1E_y** (mustard = density depletion, purple = density increase; 4.10^-4^ e.bohr^-3^).

***Figure S19***. Isosurface representations of the frontier molecular orbitals HOMO-1 to LUMO+1 (± 2.10^-2^ (e.bohr^-3^)^½^), and of the electronic charge-density difference between the first singlet (S_1_, S_2_) or triplet (T_1_, T_2_) vertical excited states and the ground state (S_0_) of **1*** (mustard = density depletion, purple = density increase; 4.10^-4^ e.bohr^-3^).

***Table S4***. Analytical data for samples of **1M**, **1E_b** and their dried derivatives*^a^*.

| **sample** | **Measured** | | **Calculated** | | |
| --- | --- | --- | --- | --- | --- |
|  | **C, %** | **H, %** | **C, %** | **H, %** | **formulation** |
| **1M** | 52.65 | 4.17 | 52.88 | 4.20 | [CuI(***P^3^OO***)Na(MeOH)_2_]_2_ |
| **1E_b** | 53.65 | 4.78 | 53.91 | 4.52 | [CuI(***P^3^OO***)Na(EtOH)_2_]_2_ |
| **1W** | 50.50 | 4.06 | 50.69 | 4.02 | [CuI(***P^3^OO***)Na(H_2_O)_2_]_2_^.^2H_2_O |
| **1W*** (**1W** vacuum dried at 120 ^o^C) | 54.05 | 3.59 | 54.12 | 3.53 | [CuI(***P^3^OO***)Na]_2_ |
| **1E*** (**1E_b** vacuum dried 155 ^o^C) | 54.14 | 3.63 | 54.12 | 3.53 | [CuI(***P^3^OO***)Na]_2_ |

*^a^* **1DME**/**1DME^vap^** could not be properly analyzed due to the loss of the DME ligand.

***Figure S20***. Thermogravimetric analyses (TGA) of methanol solvate **1M**, water solvate **1W**, its desolvated derivative **1W***, ethanol solvate **1E_b**, neat crystals **1DME**, and **1DME^vap^** generated by vapor treatment.

***Figure S21***. Normalized excitation (dashed lines) and emission (solid lines) spectra of compound **1E*** at 297 and 80 K.

***Figure S22***. Normalized excitation (dashed lines) and emission (solid lines) spectra of neat crystalline **1DME** at 297 and 80 K.

**References**

[1]	M. Beliaeva, A. Belyaev, H. Korhonen, O. Mrózek, J. Jänis, A. Steffen, I. O. Koshevoy, Acid–base interactions in luminescent silver(I) and gold(I) complexes with phosphine–phosphinate/phosphinite ligands, *Inorg. Chem. Front.* **13**, (2026), 511-524.

[2]	APEX2 - Software Suite for Crystallographic Programs, Bruker AXS, Inc., Madison, WI, USA, 2010.

[3]	CrysAlisPro*,* 1.171.43.144a, Rigaku Oxford Diffraction, 2024.

[4]	G. M. Sheldrick, SADABS-2008/1 - Bruker AXS Area Detector Scaling and Absorption Correction, Bruker AXS, Madison, Wisconsin, USA, 2008.

[5]	G. M. Sheldrick, Crystal structure refinement with SHELXL, *Acta Crystallogr. C: Struct. Chem.* **71**, (2015), 3-8.

[6]	L. J. Farrugia, WinGX and ORTEP for Windows: an Update, *J. Appl. Crystallogr.* **45**, (2012), 849–854.

[7]	O. V. Dolomanov, L. J. Bourhis, R. J. Gildea, J. A. K. Howard, H. Puschmann, OLEX2: A complete structure solution, refinement and analysis program, *J. Appl. Cryst.* **42**, (2009), 339-341.

[8]	A. L. Spek, PLATON SQUEEZE: a tool for the calculation of the disordered solvent contribution to the calculated structure factors, *Acta Cryst.* **C71**, (2015), 9–18.

[9]	F. Neese, Software update: The ORCA program system - Version 5.0, *WIREs Comput. Mol. Sci.* **n/a**, (2022), e1606.

[10]	J. P. Perdew, M. Ernzerhof, K. Burke, Rationale for mixing exact exchange with density functional approximations, *J. Chem. Phys.* **105**, (1996), 9982-9985.

[11]	C. Adamo, V. Barone, Toward reliable density functional methods without adjustable parameters: The PBE0 model, *J. Chem. Phys.* **110**, (1999), 6158-6170.

[12]	A. Schäfer, H. Horn, R. Ahlrichs, Fully optimized contracted Gaussian basis sets for atoms Li to Kr, *J. Chem. Phys.* **97**, (1992), 2571-2577.

[13]	A. Schäfer, C. Huber, R. Ahlrichs, Fully optimized contracted Gaussian basis sets of triple zeta valence quality for atoms Li to Kr, *J. Chem. Phys.* **100**, (1994), 5829-5835.

[14]	S. Grimme, J. Antony, S. Ehrlich, H. Krieg, A consistent and accurate ab initio parametrization of density functional dispersion correction (DFT-D) for the 94 elements H-Pu *J. Chem. Phys.* **132**, (2010), 154104.

[15]	I. Tamm, Relativistic interaction of elementary particles, *J. Phys. (U.S.S.R.)* **9**, (1945), 449–460.

[16]	S. M. Dancoff, Non-Adiabatic Meson Theory of Nuclear Forces, *Physical Review* **78**, (1950), 382-385.

[17]	M. E. Casida, in *Recent Advances in Density Functional Methods, Vol. 1*, World scientific, **1995**, pp. 155-192.

[18]	M. Nishio, The CH/π hydrogen bond in chemistry. Conformation, supramolecules, optical resolution and interactions involving carbohydrates, *Physical Chemistry Chemical Physics* **13**, (2011), 13873-13900.

[19]	R. Dennington, T. Keith, J. Millam, GaussView*,* 6.1, Semichem Inc., Shawnee Mission, KS, 2016.
